# Critical care management of patients following transcatheter aortic valve replacement

**DOI:** 10.12688/f1000research.2-62.v1

**Published:** 2013-02-27

**Authors:** Jesse M Raiten, Jacob T Gutsche, Jiri Horak, John GT Augoustides

**Affiliations:** 1 Department of Anesthesiology and Critical Care, Perelmen School of Medicine of the University of Pennsylvania, Philadelphia PA, 19104, USA

## Abstract

Transcatheter aortic valve replacement (TAVR) is rapidly gaining popularity as a technique to surgically manage aortic stenosis (AS) in high risk patients. TAVR is significantly less invasive than the traditional approach to aortic valve replacement via median sternotomy. Patients undergoing TAVR often suffer from multiple comorbidities, and their postoperative course may be complicated by a unique set of complications that may become evident in the intensive care unit (ICU). In this article, we review the common complications of TAVR that may be observed in the ICU, and different strategies for their management.

## Introduction

Aortic stenosis (AS) is a common heart valve disorder that affects 2–9% of the population over age 65 worldwide ^1^. Degenerative-calcific changes represent the most common etiology of AS in the elderly population ^1^. The decision of how to treat AS in the elderly depends largely on patient symptoms. In patients who are asymptomatic, despite having severe AS, the risks of surgery outweigh the benefits, and watchful waiting is prudent ^2^. However, the development of symptoms heralds the need for more aggressive treatment, often requiring surgical intervention.

Invasive therapies for AS range from balloon aortic valvuloplasty (BAV) to aortic valve replacement (AVR) via sternotomy or the transcatheter approach. In the case of traditional AVR via sternotomy, the valve may be replaced with a mechanical or bioprosthetic valve, the choice largely depending on patient age and ability to tolerate systemic anticoagulation. Over the past decade, transcatheter aortic valve replacement via the transfemoral or transapical approach (TF-TAVR and TA-TAVR, respectively) has been studied, with interest bolstered by the results of the Placement of Aortic Transcatheter Valve Trial (PARTNER trial) ^3^.

BAV is the least invasive of therapeutic choices once medical management has been exhausted, typically being performed in the cardiac catheterization laboratory. Prior to success with TAVR, BAV was considered a safe and useful option in patients who were deemed too high risk for surgery ^4^. While BAV may improve hemodynamic parameters, a high recurrence rate of valve stenosis limits the utility of the procedure. Long term survival rates are low and complications are common ^5, 6^. In the current practice paradigm, BAV may be an appropriate bridge to TAVR in patients who may be suitable candidates but need medical optimization or do not yet meet criteria for a transcatheter procedure ^7, 8^.

Evolving medical practices, including advances in surgical, anesthetic, and perioperative management techniques, have reduced morbidity and mortality associated with AVR. Overall, mortality is under 3% for all patients, but may be even lower in selected patient populations with minimal comorbidities ^9^. However, advancing patient age is creating a higher acuity patient population, many of whom are at greater risk for perioperative morbidity and mortality. A recent retrospective review of high risk patients (defined as Society of Thoracic Surgeons predicted risk of mortality of 10% or greater) undergoing isolated, primary AVR, observed an in-hospital mortality rate of 16.4% ^10^. The same study found a postoperative stroke rate of 4.4%, heart block 5%, multisystem organ failure 6.9%, pneumonia 7.5%, and dialysis 8.2%.

It is clear that traditional AVR carries considerable risk in patients with major comorbidities. In fact, 30–40% of patients with severe AS never undergo surgery due to coexisting medical conditions, heart failure, or physician and/or patient preference ^9^. Patients deemed too sick, or inoperable, are increasingly becoming candidates for TAVR, either by the transfemoral or transapical approach.

The postoperative period after cardiac surgery may include pain and mental status changes, hemodynamic instability, cardiac arrhythmias, pulmonary edema and respiratory failure, renal failure, and bleeding and coagulopathy. Advances in perioperative and surgical techniques have led to many patients being “fast-tracked”, with rapid extubation and ICU lengths of stay often less than 24 hours. However, as the patient population has become more elderly, morbidity has increased ^11^ and postoperative intensive care unit (ICU) management has become more complex. In the study previously described of high risk patients undergoing primary AVR, the median ICU length of stay was 3 days ^10^.

TAVR is less invasive than traditional AVR – no sternotomy is performed, cardiopulmonary bypass (CPB) is not necessary, and patients may be extubated in the operating room (OR). Despite its less invasive nature, over the past few years as the number of TAVRs have increased, a unique set of postoperative events and complications have been identified. While some ICU management issues are shared with patients undergoing traditional AVR, TAVR patients are predisposed to ICU concerns that the intensivist needs to recognize and manage appropriately.

## Neurological issues in the ICU

### Delirium

In the PARTNER trial, the average age at time of surgery was 83 years ^3^. It is well known that advanced age is a major risk factor for postoperative delirium (POD) after cardiac surgery. POD was not assessed in the original PARTNER trial and there is minimal data about its incidence. A small retrospective chart review found a delirium rate of 51% after TA-TAVR and 16% after TF-TAVR ^12^. In this study, TA-TAVR was associated with a significantly longer ICU length of stay (84 hours) compared to the transfemoral approach (36 hours). ICU length of stay is also an established risk factor for delirium.

POD is associated with poor outcomes including increased hospital length of stay, increased mortality, and greater nursing home placement ^13^. It is also responsible for a significant financial toll in the ICU ^14^. Given the negative consequences of ICU delirium, it is critical to quickly identify and manage through both pharmacotherapy and other interventions. While delirium may present with agitation or behavior causing self harm, hypoactive delirium is more common and is easier to misdiagnose ^14^. By virtue of their older age, comorbidities, and mandatory ICU courses, patients undergoing TAVR are high risk and should be screened for delirium and managed accordingly. There are multiple screening tools for delirium, including the Confusion Assessment Method for the ICU (CAM-ICU), and the Intensive Care Delirium Screening Checklist (ICDSC). A patient’s CAM-ICU score may be easily calculated and a patient designated as “CAM positive” if delirium is present, or “CAM negative” if they do not exhibit signs of delirium. Some ICUs, such as those at the University of Pennsylvania (Philadelphia, PA, USA), have implemented a nursing-driven protocol whereby every patient in the ICU is assessed daily for delirium as part of the daily nursing assessment. This allows early identification of the delirious patient and rapid intervention.

Antipsychotic drugs that antagonize dopamine receptors in the central nervous system are the mainstay of pharmacotherapy for ICU delirium. While haloperidol is a typical antipsychotic with a proven success record for managing delirium, newer atypical antipsychotic drugs such as quetiapine (Seroquel) and olanzapine (Zyprexa) have a lower incidence of extrapyramidal side effects ^15^. Of particular concern in the TAVR patient population, both the typical and atypical antipsychotics may be associated with a prolonged QT interval on the electrocardiogram, and an increased risk for cardiac arrhythmias. Risk factors for QT interval prolongation include female gender, polypharmacotherapy, cardiovascular disease and bradycardia, and electrolyte disorders ^15^. Unfortunately, these risk factors have a high prevalence in patients undergoing cardiac surgery.

Although at least one study in non-cardiac surgery patients has demonstrated a benefit to prophylactically treating patients at high risk for delirium ^16^, prophylactic pharmacotherapy to prevent delirium is not the standard of care in most hospitals. Maneuvers to reduce the risk of delirium, including constant orientation to time and location and maintaining a normal sleep-wake cycle should always be performed in at-risk patients in the ICU. Antipsychotic medication is usually initiated if signs of delirium develop.

### Post-operative pain

Post-operative pain after TAVR may be substantial, particularly after TA-TAVR. While TF-TAVR may be performed entirely via the femoral vessels, TA-TAVR requires a thoracotomy incision. For thoracic surgery procedures requiring thoracotomy, epidural analgesia is the current standard of care. There are numerous reports of using thoracic epidurals for cardiac surgery, with improved outcomes in terms of faster time to extubation, better postoperative analgesia, faster recovery of pulmonary function, improved participation in physiotherapy, and less depression ^17– 19^.

There are few descriptions of using thoracic epidurals for TA-TAVR. In a study of 135 patients who underwent TA-TAVR, the use of general anesthesia and thoracic epidural, compared to GA and intercostal nerve block, was associated with a significant decrease in pulmonary complications (including reintubation postoperatively), and a lower 30 day mortality in the epidural group ^20^. One case report describes the use of epidural anesthesia for TA-TAVR in a patient with severe obstructive lung disease ^21^. The patient was awake throughout the procedure and transferred to the post-anesthetic care unit and step-down unit postoperatively, bypassing the ICU entirely. TF-TAVR has also been performed in awake patients – a comparison of GA versus local/regional anesthesia with ileoinguinal-iliohypogastric blocks found local/regional anesthesia was associated with a shorter operative procedure and hospital length of stay ^22^. The rate of complications was not significantly different between groups.

While the minimal data evaluating epidural use during TAVR seems to be associated with favorable outcomes, the risks most notably include bleeding and epidural hematoma formation. Although patients are systemically anticoagulated intraoperatively, the degree of anticoagulation is generally less than that required for CPB. As mentioned above, there are numerous reports of the safe use of epidurals in patients anticoagulated for CPB. The use of other neuraxial monitors, specifically lumbar drains, are routinely employed in patients undergoing thoracoabdominal aneurysm repair, in which a high degree of systemic anticoagulation is necessary.

### Transient ischemic attack and stroke

Stroke is probably the most feared neurological complication following aortic valve surgery. The incidence of stroke after traditional AVR in all patients is approximately 1.6% ^23^, but may be greater in high risk patients and those with previous coronary artery bypass surgery ^24^. Stroke is a major source of morbidity in patients following TAVR, with an incidence of 2.4–9.1% after TF-TAVR and 1.5–6.7% after TA-TAVR ^24^. In a recent study of 31 patients who underwent TAVR, diffusion-weighted MRI identified new cerebral infarcts in 77% of patients postoperatively ^25^. The authors identified increased severity of aortic atheroma as a risk factor for new cerebral infarcts.

Neurologic events following TAVR peak in the first week postoperatively. In an analysis of patients enrolled in the PARTNER trial, 12/31 events (stroke or TIA) occurred between postoperative days 0–2 ^24^. In a study of 253 patients who underwent TAVR (who were not part of the PARTNER trial), the risk of stroke or TIA was greatest in the first 24 hours after surgery ^26^.

While perioperative and intraoperative hypotension may be responsible, neurologic events occurring in the first 24 hours are likely related to embolization of calcium and debris, or thrombi that may form on wires and surgical devices intraoperatively ^24, 26^. Indeed, a smaller aortic valve area index is associated with a greater risk of early stroke, possibly due to increased propensity for calcium embolization ^24^. New devices are in development to reduce the incidence of cerebral emboli, including the SMT Embolic Deflection Device, which acts as a filter in the aortic arch, reducing emboli to the brain ^27^.

Infarcts affecting the distribution of the middle cerebral artery, the posterior circulation, or involving multiple sites are often embolic in nature, and are characteristic of the cerebral infarcts seen immediately after TAVR ^26^. We believe knowledge of the clinical presentation of embolism to these vessels is critical in order to rapidly diagnose and treat any events. In recognition of the risk of embolic stroke, in the absence of significant postoperative bleeding, anticoagulation is usually initiated on postoperative day 1. Dual antiplatelet therapy (aspirin and clopidogrel) may be used, or aspirin and warfarin if a patient is already taking warfarin for concomitant atrial fibrillation. Although there are no absolute guidelines, anticoagulation is often continued for several months following surgery.

## Cardiac issues in the ICU

### Heart rhythm

Preexisting cardiac conduction system disease is common in patients undergoing AVR, and in one study it was identified in 23% of patients preoperatively ^28^. Baseline conduction abnormalities (particularly bundle branch blocks (BBB)) have been identified as a major risk factor for post-procedural permanent pace maker (PPM) ^28, 29^. Depending on the type of valve (Sapien versus CoreValve), post TAVR PPM is reported in 1.8–8.5%, and 19.1–42.5%, respectively ^9^. Anatomical compression on the conduction system by the prosthesis is likely a major contributing factor to postoperative conduction defects.

New onset atrial fibrillation (NOAF) after TAVR is also common and was identified in 31.9% of patients in one prospective study, at a median time of 48 hours postoperatively ^30^. NOAF was more common with a larger left atrial size and with TA-TAVR. While associated with a higher risk of stroke, NOAF did not increase mortality in this study.

Cardiac pacing wires are used intraoperatively to allow rapid cardiac pacing during valve deployment. These wires may be left in place afterwards in patients who experience heart block during the procedure or who have risk factors for arrhythmias postoperatively (preexisting BBB, large left atrial size). While patients who develop heart block during the procedure may be paced in the ICU until PPM can be placed, a backup mode may be useful in patients at risk for arrhythmias. Avoidance of negative chronotropes (beta blockers, digoxin, etc) is prudent in patients with preexisting BBB at risk for worsening arrhythmias.

### Other cardiac complications

While cardiac complications are common in patients undergoing TAVR, the vast majority are intraoperative events that are diagnosed and treated before the patient arrives in the ICU. Complications seen intraoperatively include coronary artery occlusion from malposition of the graft, myocardial infarction, tamponade, rupture of aortic root or annulus, mitral valve apparatus injury, excessive bleeding, valve migration, and paravalvular leak. Signs of apical myocardial infarction may be seen on electrocardiograms postoperatively and are often related to apical puncture to facilitate valve placement in TA-TAVR.

Labile hemodynamics are common in the immediate postoperative period following TAVR. Left ventricular hypertrophy and diastolic heart disease often make patients very volume responsive, and hypotension is often responsive to volume resuscitation. Hypertension that is not pain related may be managed with nicardipine, a rapidly titratable calcium channel blocker. While there are no well established guidelines for BP management postoperatively, we believe targeting a mean arterial pressure of 60–80 is reasonable.

## Pulmonary considerations in the ICU

Patients undergoing TAVR are considered inoperable by traditional criteria. Coexisting pulmonary disease, most notably chronic obstructive pulmonary disease (COPD), is common in this patient population and was present in 41% of patients randomized to TAVR in the original PARTNER trial ^3^. Of note, 21% of patients had oxygen dependent COPD. The combination of severe lung disease, postoperative pain from sternotomy, and prolonged time under anesthesia in patients undergoing traditional AVR may contribute to difficulty with ventilator weaning in the ICU. In contrast, we believe many patients undergoing TAVR may be fast tracked and extubated at the end of the procedure, appreciable largely to the absence of sternotomy and less postoperative pain, shorter surgical times and less exposure to anesthesia.

The need for reintubation in the postoperative period may be related to two primary factors-pain, particularly following TA-TAVR, and pulmonary edema. LVH and diastolic heart disease that often accompanies AS may necessitate significant volume resuscitation to maintain stable hemodynamics. As this volume equilibrates in the early postoperative period, pulmonary edema and effusions may develop. This fluid buildup, particularly pleural effusions, may be more clinically significant than following traditional AVR, as chest tubes are not usually placed for TF-TAVR, and only one may be present after the TA-TAVR. Thoracentesis to remove pleural fluid may be necessary as the need for higher cardiac filling pressures to maintain hemodynamics may prevent aggressive diuresis.

## Renal failure in the ICU

Given the high acuity of patients undergoing TAVR, it is not surprising that a significant percentage suffer from renal insufficiency. Five percent of patients randomized to TAVR in the PARTNER trial had a baseline creatinine > 2mg/dL ^3^, and almost 50% of patients in another series had preoperative renal failure ^31^. The requirement for intra-arterial contrast medium predisposes patients to acute (ARF), or acute on chronic renal failure postoperatively. In a series of 110 TAVR patients, *new onset* post-procedure acute kidney injury (AKI) was found in 9% of patients, while 35% of patients with preexisting renal insufficiency experienced acute on chronic failure ^31^. In the majority of cases, ARF was mild and transient, with only one patient requiring hemodialysis. Perioperative red blood cell transfusion has been associated with AKI in TAVR patients ^32^.

Numerous studies have sought to identify ways to prevent and treat contrast-induced nephropathy (CIN). Techniques include ensuring excellent perfusion pressure, administration of *N*-acetylcysteine, hydration with normal saline or sodium bicarbonate, and post-procedure hemofiltration. While the data is plentiful on the topic, the results are inconsistent regarding the optimal preventive strategy for CIN. Aggressive hydration with sodium bicarbonate carries risks of pulmonary edema and hypercarbia, particularly in the TAVR patient population where heart failure and COPD are common. In patients with significant cardiopulmonary comorbidities, avoiding hypotension and minimizing contrast exposure may be the most prudent ways to decrease the risk of CIN.

## Vascular access complications

Vascular complications are common after TAVR and occurred in 30% of patients in the PARTNER trial (16% were major complications) ^3^. In a prospective study of 130 TF-TAVR patients, vascular complications were predicted by center experience, femoral calcification, and the sheath to femoral artery ratio score (SFAR) ^33^. In a review of 101 patients undergoing TF-TAVR, vascular access complications occurred in 32%, and 10% required surgical repair ^34^. Vascular complications include retroperitoneal hemorrhage, femoral or iliac artery dissection, and development of a femoral pseudoaneurysm.

Some complications may become apparent intraoperatively. However, initial recognition of a vascular access complication is often detected in the ICU postoperatively. Proper techniques must be ensured with removal of any femoral arterial sheath. Pressure at the puncture site must be held for an adequate length of time (usually 3–5 minutes for each French size of the catheter), in order to achieve hemostasis. Inadequate pressure can result in pseudoaneurysm or hematoma formation. At our institutions, we remove femoral access sheaths in the OR before the patient is transported to the ICU.

The presence of a high femoral arterial stick (above the inguinal ligament) may first present in the ICU with the development of a retroperitoneal bleed when the line is removed, despite adequate pressure being held. We believe hypotension presenting in the hours after arterial line removal should trigger a rapid workup for possible retroperitoneal hemorrhage. Non-contrast computed tomography (CT scan) is the study of choice to identify a retroperitoneal bleed, but in the presence of unstable hemodynamics, abdominal tenderness and a newly removed formal arterial catheter, it may be prudent to proceed directly to the OR for surgical exploration.

In a retrospective review of more than 9000 patients undergoing femoral artery catheterizations, presenting signs of retroperitoneal hematoma included suprainguinal tenderness in 100% of patients, severe back pain in 64%, and femoral neuropathy in 36% ^35^. The diagnosis may be more difficult if the patient is sedated or mechanically ventilated, and a high index of suspicion is needed.

## Conclusion

TAVR is an innovative method to treat aortic valve disease in high risk patients. Its minimally invasive nature eliminates the need for sternotomy, CPB, and reduces procedural and anesthesia time. Nevertheless, TAVR is a major surgical procedure with considerable morbidity and mortality, and intensivists caring postoperatively for these patients must be able to treat the immediate postoperative complications. Prompt recognition of postoperative neurologic events, cardiac arrhythmias, renal failure, vascular complications and hemorrhage are critical to improve patient safety and outcomes.
