# Supplementary material for: Critical care management of patients following transcatheter aortic valve replacement
Source: F1000Res. 2013 Feb 27;2:62. [Version 1] doi: 10.12688/f1000research.2-62.v1 (PMC3752734; doi:10.12688/f1000research.2-62.v1)
Supplement: Peer review history

# Peer review: Referee response for version 1

**Published:** 2013-03-04

**Authors:** Hannah Wunsch

**Affiliations:** Assistant Professor of Anesthesiology and Critical Care, Columbia University, New York, NY, USA

This review article addresses the post-operative management of a relatively new technique for valve replacement. The topic is important and the authors nicely review the range of possible post-operative complications.

I have a few small concerns. The first is that the majority of the topics covered are applicable to all cardiac surgery patients. I think it would be helpful for the authors to highlight better which are the issues that are truly specific to these patients (i.e. retroperitoneal hematoma, and possible management of severe COPD) and which are general to valve surgery (e.g. risk of stroke).

Second, there are a number of statements that should either be changed, or referenced:

-There should be references for the two delirium screening systems described. 

-The authors state that haloperidol has a “proven success record for mgmt. of delirium”.  I am not aware of good studies that prove this; it should either be referenced or removed.

-Studies on prophylaxis for delirium are conflicted. The statement in the paper implies that prophylaxis should be standard of care in hospitals. I suggest tempering this statement.

-“Epidural anesthesia is the current standard of care” either should be referenced or removed, as, again, this implies that physicians who are not supplying epidurals to these patients are failing to provide appropriate care.

-“Dual antiplatelet therapy (aspirin and clopidogrel) may be used, or aspirin and warfarin if a patient is already taking warfarin for concomitant atrial fibrillation” should be referenced, or it should be made clear that there are no guidelines and this is purely the opinion of the authors.

-“We believe targeting a mean arterial pressure of 60–80 is reasonable,” would also benefit from a reference, or perhaps include some additional caveat regarding taking into account a patient’s preoperative blood pressure. 

-Second paragraph on renal failure – data “are” plural. Currently says “data is”

I have read this submission. I believe that I have an appropriate level of expertise to confirm that it is of an acceptable scientific standard.

# Peer review: Referee response for version 1

**Published:** 2013-03-04

**Authors:** Hynek Riha

**Affiliations:** Department of Anesthesiology and Intensive Care Medicine, Institute for Clinical and Experimental Medicine, Prague, Czech Republic

This is an excellent review paper covering all important issues encountered during postoperative course after transcatheter aortic valve replacement (TAVR). The review is important as the number of these procedures performed worldwide is quickly increasing. Management strategies after TAVR described in the paper are supported by recent references.

I have read this submission. I believe that I have an appropriate level of expertise to confirm that it is of an acceptable scientific standard.
